# Surgical Treatment of Pediatric Scoliosis: Historical Origins and Review of Current Techniques

**DOI:** 10.3390/bioengineering9100600

**Published:** 2022-10-21

**Authors:** Andrew M. Block, Lisa M. Tamburini, Francine Zeng, Michael R. Mancini, Casey A. Jackson, Christopher L. Antonacci, Owen P. Karsmarski, John W. Stelzer, Ian J. Wellington, Mark C. Lee

**Affiliations:** 1 Department of Orthopaedic Surgery, University of Connecticut, Farmington, CT 06032, USA; 2 Department of Orthopaedic Surgery, Connecticut Children’s Medical Center, Hartford, CT 06106, USA

**Keywords:** scoliosis, growing rods, tethering, deformity, correction

## Abstract

The treatment of scoliosis has been explored and debated in medicine since the first recorded texts. Scoliosis treatment has shifted over time from external modalities, such as traction and bracing, to internal stabilization techniques that leverage surgical advances. Surgical fixation constructs can generally be separated into two different modalities: dynamic vs. static constructs. For skeletally immature individuals with progressive deformities, surgical options range from traditional or magnetically controlled growing rods to vertebral body staples or tethering. For individuals who have reached skeletal maturity, many devices have been developed that provide static length constructs. Understanding the surgical options available is critical for the appropriate management of this varied patient population. With this article, we sought to provide a summary of past and present techniques and devices used in the treatment of scoliosis.

## 1. Introduction

The treatment of scoliosis has been discussed for thousands of years, with descriptions of the disease and proposed remedies offered in the earliest recorded texts. The very first reference to successful scoliosis treatment is found in the Srimad Bhagwat Mahapuranam, an ancient Hindu religious text written between 3500 and 1800 BCE. The manuscript describes the Lord Krishna curing a devotee with a multi-directional spinal deformity by applying traction under the patient’s chin [1]. In ancient Rome, mechanical traction was the treatment modality described by famous philosopher-physicians Hippocrates and Galen, along with the devices used to apply the traction [2]. 

Treatment of scoliosis continued to advance through the years, well beyond the religious or philosophical realm, and now rests comfortably in the very core of orthopedics. Indeed, the word orthopedics translates to “straight child,” as coined by Frenchman Nicholas Andry in the 1700s [2]. Today, scoliosis is defined as an abnormal curvature of the spine in the coronal plane and is noted to occur in both skeletally mature and immature individuals. As the field of orthopedics expanded to develop the basic principles that surgeons are familiar with today, scoliosis treatment shifted away from the ancient focus on external traction and instead began to focus on developing internal surgical treatment techniques. The very first reported case of a successful surgical correction utilizing wire instrumentation was reported in 1891 by Berthold Hadra [3]. The first use of bony fusion was described by Russell Hibbs, who utilized autologous bone chips from laminae in 1911 [4]. 

Subsequently, the techniques and implants used to correct scoliosis have evolved quickly to allow powerful deformity correction while imparting the least amount of morbidity. For the pediatric patient population with varying degrees of remaining growth, two major categories of surgical techniques for scoliosis correction have emerged: dynamic and static constructs. Given the range of etiologies for scoliosis and that the deformity exists within a diverse patient population, an understanding of the numerous available surgical techniques and implants and their appropriate clinical applications is critical to optimize surgical outcomes for patients. With the current work, we sought to summarize the available surgical techniques and implants and provide both a historical perspective and contemporary outcome data. 

## 2. Static Instrumentation

### 2.1. Early Rods 

For those patients without significant residual growth, a fixed construct that can prevent progression of the curve into adulthood has been the standard treatment for scoliosis since Paul Harrington’s work in the 1950s. Harrington rods were born out of necessity to treat patients with polio who were unable to tolerate cast correction techniques due to cardiopulmonary restrictions [5]. Harrington’s system was designed to provide distraction of the spine, with handmade, threaded compression rods and a distraction bar ratcheted to hooks on the transverse process or lamina. Following surgery, immobilization was then used for a period of 6 to 9 months. Although initially implanted without concurrent fusion, it became clear that fusion was needed, as many patients lost correction by 6 months to 1 year with hardware failure [6]. As fusion was added and instrumentation was refined, results improved and Harrington rods became the standard of care [6]. Unfortunately, while distraction allowed for maintenance of the fixation in the coronal plane, subsequent loss of the sagittal profile of the spine resulted in poor long-term outcomes. Nearly all patients in the long term had significant back pain, a constellation of symptoms now called “Flatback Syndrome” [7]. Additionally, complications, such as pseudoarthrosis with rates as high as 40%, hook dislodgement, and general hardware failure, were noted [8].

**Figure 1:**
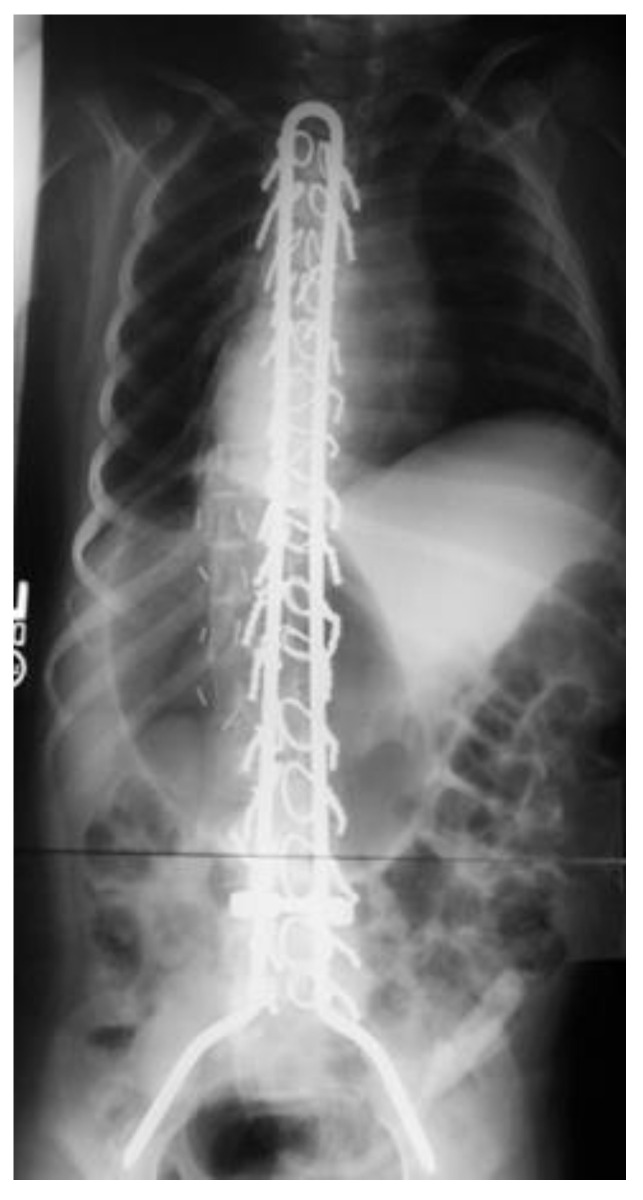
Anterior-posterior radiograph of a Unit rod construct using Luque wiring used for the treatment of a patient with neuromuscular scoliosis.

In the 1970s, Luque took Harrington rods a step further by expanding the number of points of fixation. Based on lateral bending as the predominant corrective force, the Luque rods were pre-bent L-shaped rods placed on either side of the spine (Figure 1). The L-shape design was intended to prevent migration of the bar superiorly or inferiorly. A sublaminar wire was passed through the spinal canal at each vertebral level and then wrapped around both the lamina and the bar [9]. Given the rods laterality, the convex rod could be placed first and used to lever the spine straight in the frontal plane with superior rotary control. By allowing for multiple points of fixation, corrective forces could be more evenly distributed. Outcomes compared favorably to the Harrington rods. Given that fixation occurred at each level, failure of bone or wire did not compromise the entire construct [10]. However, a central drawback was the risk of neurologic complications given the amount of cord manipulation required for wire implantation and revision, if required [9,10,11].

As an alternative spine instrumentation technique, pedicle screw fixation emerged. In 1970, Roy-Camile described pedicle screw fixation in combination with posterior plates and reported near 100% success with lumbosacral fusions [12]. Variations of a pedicle screw and plate system were offered until the mid-1980s, when Cotrel and Dubousset popularized a system using lumbar pedicle screws and thoracic or lumbar hooks, along with dual rods, for scoliosis correction [13]. Using segmental fixation with screws or hooks, concave rods to create a distraction force, and a rod rotation maneuver to translate the spine, the system offered excellent stability and new techniques for deformity correction. The system decreased the neurologic risks associated with the Luque technique and required no postoperative casting and bracing. Unfortunately, the constructs were still quite rigid, with pseudoarthrosis rates as high as 33% and pull-out rates of 44% [14]. Screw fixation continued to replace hook and wire fixation in the lumbar spine as both biomechanical and clinical studies demonstrated that screws better resist tensile forces [15,16]. Screws eventually replaced hook and wire constructs in the thoracic spine, with Suk demonstrating that pedicle screw instrumentation in the thoracic spine for scoliosis correction was feasible, safe, and effective [17]. Modern day deformity correction leverages pedicle screw instrumentation (discussed later in this manuscript) for its modularity, correction power, ease of engagement to subsequent spanning rods, and the ability to gain stable fixation without the need to preserve the posterior elements of the spine.

### 2.2. Pelvic Instrumentation 

A modification to Harrington and Luque rod instrumentation that significantly improved the rates of pseudoarthrosis was the addition of pelvic fixation. Fixation across the lumbosacral junction was mechanically superior, resisting the flexion and cantilever forces that can be responsible for pseudoarthrosis [11]. For those patients with neuromuscular disorders and/or those who are non-ambulatory, this is especially effective. In addition, pelvis instrumentation allows control of pelvic obliquity, a primary concern in managing non-ambulatory patients with spine deformity. 

**Figure 2:**
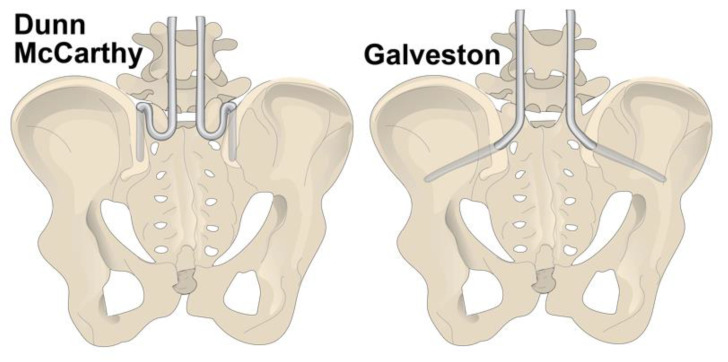
Galveston and Dunn-McCarthy methods for pelvic fixation of lumbar instrumentation.

One method of adding pelvic fixation to a Luque rod construct was to bend an extension into the distal end of the rod to engage the ilium through the posterior superior iliac spine (PSIS) [18]. The rod extension was termed the Galveston technique (Figure 2). Unfortunately, since the rods crossed the mobile sacroiliac joint, the rods were able to move independently in the coronal plane and “windshield wiper,” dislodging the fixation points and resulting in non-union [19,20]. Another significant drawback was implant prominence, given the proximity of the PSIS to surface anatomy [18].

The unit rod (colloquially called U-rod) is a variation on Luque-Galveston fixation that was found to be more stable than the individual Luque rods. Developed in the late 1980s by Bell et al. [21], the construct was a single, continuous U-shaped rod, joined along the cephalad end, with two distal rod ends bent to engage the bilateral iliac wings. The connection eliminated the risk of two unlinked Luque rods rotating independently of each other [22]. In one of the largest studies of U-Rods in patients with cerebral palsy, Tsirikos found excellent correction of the scoliosis and pelvic obliquity, with a mean improvement of 68% (76 to 25 degrees) and 71% (17 to 5 degrees), respectively [23]. Unfortunately, many of the neurologic risks of the Luque technique were still present in the Unit Rod. Additionally, given that the rod is a fixed construct, the insertion of the iliac components presented technical difficulty for those patients with severe lumbar hyperlordosis [24].

Another form of pelvic fixation is found in the Dunn-McCarthy method. After releasing iliolumbar ligaments and soft tissues off the sacral ala, two pre-bent S-shaped rods (Dunn-McCarthy hooks) are placed over the sacral ala and sit below the transverse processes of L5 and the ilium (Figure 2). The longitudinal rods can then be seated adjacent to the bone. Most often used in non-ambulatory patients with significant thoracolumbar or lumbar kyphosis, this was designed in response to the osteomalacia and thin ilia found in neuromuscular patients [25]. While Dunn-McCarthy hooks provide adequate fixation without crossing the sacroiliac joint, its primary drawback was L5 nerve root impingement and interference with the lumbosacral plexus in 14% of patients [26].

Modern refinement of the Galveston technique utilizes modular screws placed into the ilium. Screws are inserted into the posterior iliac spine and are aimed towards the anterior inferior iliac spine. With more bone to work with, longer screws may be used, which increases the screws’ purchase and prevents pullout. Biomechanical studies have shown that this trajectory allows for a different plane of fixation and offered three times stronger fixation compared to the original Galveston technique [27]. The screw modularity facilitates rod engagement and fixation point insertion. Downsides of this fixation method are potential damage to structures in the sciatic notch, rod disengagement, and deep infections [28,29]. In addition, an outrigger or screw head extension was often required to connect to the primary rods given the lateral starting point of the screws. A variation on modular iliac screws was offered by Sponseller and Kebaish, titled S2-Iliac fixation, where the screws begin lateral to the S2 foramen and traverse the SI joint [30]. The starting point was advantageous in being directly in line with the lumbar fixation and obviated the need for screw head extensions to the primary rod. 

### 2.3. Fixation to the Spine 

#### 2.3.1. Screws

The method of fixation to the spine has also seen robust variation and innovation. The major subtypes of attachments have been screws, hooks and bands. Pedicle screw fixation is the mainstay of modern instrumentation and, as previously reviewed, has undergone numerous cycles of design change and improvement to facilitate insertion, expedite rod seating, and improve pull-out strength.

First, there have been significant modifications to the screw head and body relationship. Early pedicle screw constructs were all monoaxial, with a head fixed to the body. While this has shown increased rotational leverage during de-rotation maneuvers [31], the rod may not always be easily seated in the screw and can result in loosening or rod–screw interface failure [32]. Today, pedicle screw fixation options still include a fixed head construct but also have expanded to include heads with a single axis of motion (monoaxial) or polyaxial screws. A hybrid construct composed of both monoaxial and polyaxial screws is a popular construct. 

Next, the screw material and coating has been a significant topic of investigation with regard to pull-out strength and durability and is beyond the scope of this review [32]. Commonly identified complications associated with pedicle screws have included difficulties with placement and potential for neurologic or vascular injury, screw loosening or pullout, and screw fracture [32]. 

The bulk of the current research focuses on the optimal insertion technique for pedicle screws, with significant interest in computer-assisted or robotic navigation systems. A recent systematic review found that there is insufficient evidence to suggest either navigated or free-hand techniques are superior in spine deformity correction [33].

#### 2.3.2. Hooks

Modular hooks used for spinal fixation were developed in the 1990s, concurrently with dual-rod constructs. Hooks are classified according to their directionality or anatomic location. Hooks rely on the presence of intact posterior elements or transverse processes. The direction of the hooks are either “up-going” or “down-going,” depending on the direction of the hook’s “throat” or portion that is used to capture the anatomic bony landmark (e.g., vertebral pedicle, lamina, or transverse process). The segment that engages the rod (“tulip”) is similar to pedicle screw heads and can be straight or offset from the throat to accommodate the distance from the bony articulation to rod. A major advantage of hooks are their relatively favorable pull-out strength with built-in “flexibility”, since they are not rigidly fixed to the spine. These hook properties seem to be optimal for creating less rigid fixation at the upper and lower ends of instrumentation. These “soft landings” at the end of the posterior spinal constructs are correlated with a decreased rate of proximal junctional kyphosis [34]. When comparing costs, hooks are significantly cheaper than pedicle screws [35]. 

One major disadvantage of hooks is that they are unable to achieve multi-column vertebral control for manipulation of the spine, since they can only apply force to the posterior elements. Additionally, hooks are fixed in a dynamic fashion and have to be tensioned with compression and distraction through the posterior rod to properly engage the bone. If not properly seated, hook loosening can occur. Finally, there is increased risk of neurologic injury during placement with certain hook locations, specifically sublaminar hooks as they are in closer proximity to neural elements [36].

#### 2.3.3. Wires and Bands 

Stainless steel or titanium sublaminar wires were described earlier as a component of the Luque fixation technique. However, the rigidity of the wire posed potential neurologic injury risk during placement and tightening of the rod [37]. Flexible sublaminar bands were developed to make such sublaminar fixation safer and have become one of the primary tools used in adolescent idiopathic scoliosis and neuromuscular scoliosis surgery. Mazda et al. [38] first described this technique in 2009 as an advancement to sublaminar wires for posteromedial spine translation. Sublaminar bands are made from acrylic or polyester material to make them more flexible and reduce the risk of injury to the spinal cord. In general, sublaminar bands are placed with a technique similar to sublaminar wires and sequentially tensioned against rods to facilitate a technically simple method of posteromedial spine translation [39]. Constructs can be either band-only or hybrid constructs with pedicle screws, hooks, or sublaminar wires at the cephalic level of the fixation [40,41]. Band-only constructs are primarily used for non-ambulatory patients with neuromuscular scoliosis and hybrid constructs are used for ambulatory patients with adolescent idiopathic scoliosis or neuromuscular scoliosis or for non-ambulatory patients with neuromuscular scoliosis [42]. 

The ability of sublaminar bands to progressively tension the spine offers an enhanced band-to-bone contact area, decreased laminar fracture risk, and increased corrective forces [43]. Furthermore, sublaminar bands have been shown to provide suitable correction in the coronal and sagittal planes for both adolescent idiopathic scoliosis and neuromuscular scoliosis patients [44,45,46]. Sublaminar band hybrid constructs have been demonstrated to restore 66–71% of the main thoracic curve with only 3–4% loss of correction at 2 years after operation [47]. Additionally, Canavese et al. reported an average of 24° improvement in thoracic kyphosis and that 85% of patients had a sagittal alignment within normal limits after fusion [42]. Sublaminar bands appear to be a safe and effective fixation option in the management of adolescent idiopathic scoliosis and neuromuscular scoliosis. 

## 3. Dynamic Instrumentation

### 3.1. The Luque Trolley System

The surgical correction of spinal deformities in children poses a unique challenge given the potential for continued spine growth. While static constructs can be effective in the correction of deformities in older children and young adults, such techniques can lead to trunk growth arrest, premature loss of motion, and recurrent deformity when applied to patients with significant residual spine growth. For young patients with spine deformities, a prevailing theory suggests that surgical techniques that allow spine growth may prevent the complications of a static construct while controlling the spine deformity.

One of the first dynamic systems was the Luque trolley system in 1977 [9]. This system was a combination of two L- or U-shaped rods (discussed below) attached by sublaminar wires to the spine of a skeletally immature patient without fusion. The construct served to guide spine growth (with the wires gliding along the rod, similar to a trolley) while still providing correction of the scoliosis and had a goal of maintaining trunk growth while minimizing repeated surgical interventions. Unfortunately, subsequent case studies did not demonstrate maintained correction in the majority of patients and many patients experienced hardware failure that required revision [48,49]. Additionally, spinal growth was often inhibited, likely due to the periosteal dissection needed to place the sublaminar wire anchors. The Luque trolley system has since been superseded by other dynamic modalities. 

### 3.2. The Shilla System 

The Shilla system leverages a similar concept to that of the Luque trolley’s growth along a rod. A fusion was created at the apex of the scoliosis and pedicle screws that allowed sliding along the rod were used at the cranial and caudal ends of the construct [50]. After a mean follow-up period of seven years, McCarthy and McCullough showed that the average initial preoperative curve of 69° (range of 40–115°) was corrected to 38° (range of 16–74°) [51]. In addition to favorable curve correction, the technique avoids repeated open lengthening surgeries that were seen in other modalities. Unfortunately, there existed a high rate of implant failure and wound complication that required revision surgery [51,52,53]. Occasionally, the caudal and cephalad screws slid off the rod and required surgical revision. Several technique modifications using different compression models or different screw configurations were proposed to optimize correction while reducing complications [54]. However, these techniques were met with varying degrees of success and none of which have gained widespread adoption. 

### 3.3. Traditional Growing Rods

Traditional growing rods provide an alternative option for the surgical correction of scoliosis while also allowing for the continued growth of the child. The pediatric Isola rods were originally used in both single and dual growing rod constructs. Hooks or screws are used as foundations at the cephalad and caudal ends of the construct. The Isola rods were stainless steel and contoured the patient’s kyphosis [55]. In the single rod construct, the caudal end of the rod remains long to allow for interval lengthening. This construct is manually lengthened in intervals until definitive posterior spinal fusion can be performed, typically at age 10 for girls and age 12 for boys. Cessation of lengthening was also determined by the child’s size, curve magnitude, health, and ability to tolerate additional lengthening [56,57]. In the dual-rod construct, each rod is made up of two sections connected by a tandem connector which allows for lengthening [55,56]. These rods are lengthened by loosening set screws at the tandem connector and applying distraction every 6 months until the patient is age-appropriate for static spinal fusion. Both single and dual traditional growing rod techniques were studied starting in the early 1990s. Thompson et al. compared three treatment groups: single growing rods with short anterior and posterior apical fusion, single growing rod without fusion, and dual growing rods. When comparing the preoperative curve size to the final curve size at the end of treatment for each of the previously noted groups, the percent correction was 23% ± 22, 36% ± 23, and 71% ± 22, respectively [58]. Additionally, they reported complication rates of 80, 19, and 29% in the single rod with fusion, single growing rod, and dual growing rod groups, respectively [58]. In the long term, it was found that those who more frequently underwent lengthening (≤6 months) had a higher annual growth rate and greater correction of scoliosis when compared to those who were less frequently corrected [59]. Common complications from this treatment technique were broken rods, hook displacement, and infections [56,57,58]. Overall, traditional growing rods remain a viable option for management of the young spine.

### 3.4. Magnetically Controlled Growing Rods 

While traditional growing rods were generally effective, the need for repeated surgeries and open rod lengthening motivated the development of remotely expandable instrumentation. One of the earliest such devices was the Phenix implant. Originally used in limb reconstruction following tumor resection in children [60], the implant was composed of one titanium tube within a polymer tube, with a compressed spring between the two. An applied external magnetic field would induce a current that would melt a polyethylene insert, allowing for progressive expansion of the tensioned spring and resulting in elongation of the implant [61].

**Figure 3:**
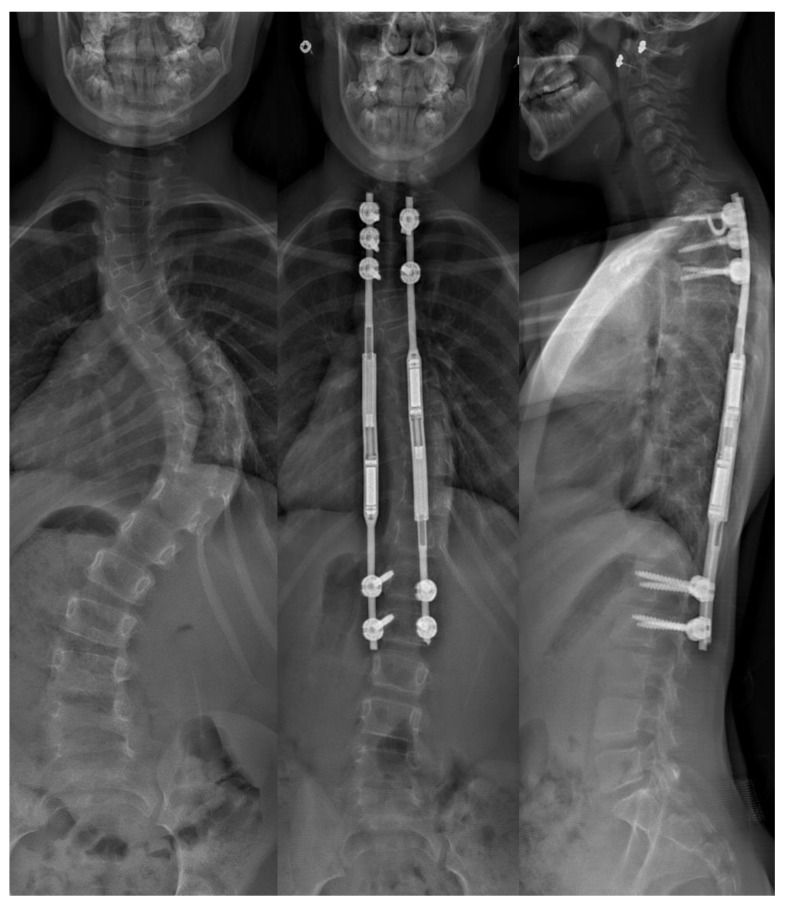
Left: preoperative of large magnitude scoliosis, Center: postoperative anterior-posterior radiograph, Right: lateral radiographs following the application of two magnetic growing rods with significant improvement of the curvature.

The most commonly used modern remote lengthening systems are magnetically controlled growing rod (MGR) devices. Magnetic growing rods function similarly to traditional growing rods. Hook or screw fixation is obtained at the cephalad and caudal segments of a deformity and either single or dual rods are used to connect the fixation points (Figure 3). Rods are lengthened in a physician’s office using a handheld magnetic device, which rotates an actuator within the rod and causes lengthening [62,63]. MGRs have grown significantly in popularity since their introduction, comprising < 5% of all growing rods in 2007–2008 and increasing to 83% in 2016–2017 [64].

While MGRs have a high initial cost, due to the price of the implants, they achieve cost equivalency to traditional growing rods at 3 years from implantation, with further cost savings beyond this point [65,66]. These cost savings are attributed to the avoidance of multiple surgeries for implant lengthening. When compared to traditional growing rods, clinical outcomes with MGR have been promising, with similar rates of proximal junctional kyphosis and decreased rates of superficial and deep infections [67,68]; however, mechanical failure that requires revision may be more common [69]. Additionally, actual MGR distraction lengths were demonstrated to be 14% less than the programmed target length, which calls into question the ability of these constructs to reliably grow the spine [70]. Regardless, the clinical benefit of these rods in avoiding repeated surgeries may outweigh the incongruence between programmed and actual lengthening.

### 3.5. Vertical Expandable Prosthetic Titanium Rib (VEPTR) 

**Figure 4:**
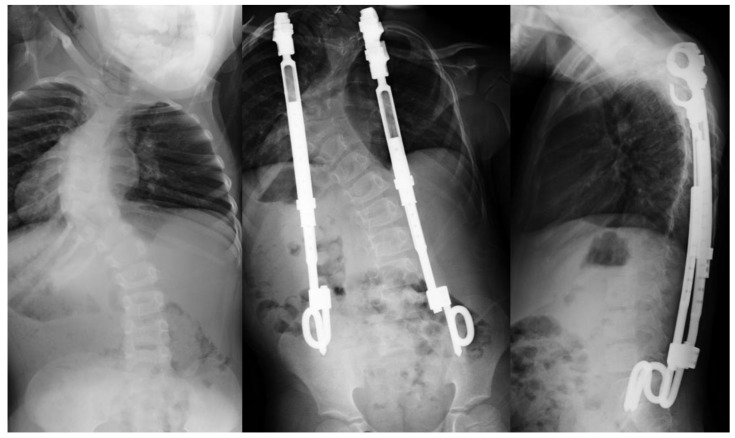
Left: preoperative of large magnitude scoliosis, Center: postoperative anterior-posterior radiograph, Right: lateral radiographs following the application of two VEPTR rods.

The vertical expandable prosthetic titanium rib (VEPTR) is a dynamic growth technique that was developed in 1987 and became FDA approved in 2004, primarily for the treatment of thoracic insufficiency syndrome [71]. The VEPTR concept is based on the expansion of the thorax by rib distraction on the concave side of the spinal curve, with concurrent impact on the spine deformity (Figure 4). Patients undergo consecutive distractions every 4–6 months until skeletal maturity is achieved [72]. The superior hooks of the titanium devices were originally secured to the third through fifth ribs, depending on the upper end vertebra of the scoliotic curve. The caudal fixation site varied based on the severity of the curve and disease process but could be placed on ribs, spine, or pelvis (Figure 4). The primary indication for the use of VEPTR is thoracic insufficiency syndrome in skeletally immature patients, defined as the thorax’s inability to support normal respiration and/or lung development [73]. These syndromes are anatomically diverse and may manifest as absent ribs, multiple rib fusions and/or early onset scoliosis of congenital or neurogenic origin, with or without rib anomalies [74]. Most outcome studies have demonstrated that the VEPTR technique can improve thoracic insufficiency syndrome and correct scoliosis deformity but the percentage of improvement in degrees of deformity has varied between 17 and 59% [75]. Compared to traditional growing rods, the control of sagittal plane deformity and pelvic obliquity was comparable but the correction of coronal plane deformity was less [76]. The majority of complications from the use of VEPTR consist of infection, secondary to repeat distraction operations, which is similar to traditional growing rods, making magnetically controlled growth rods preferable by comparison [71].

### 3.6. Staples 

Vertebral body stapling (VBS) were introduced in 1951 to modify the growth of the spine with an internal restraint along the convexity of the deformity, similar to stapling across the physes of long bones to treat angular limb deformity. Nachlas et al. created and corrected unilateral rotatory lumbar scoliosis in canines by placing staples across the vertebral physeal end plates [77]. In 1954, Smith et al. published a case series of stapling across two contiguous vertebral bodies in three children. Unfortunately, the subjects were relatively mature with severe curves, resulting in little curve growth modulation and hardware failure [78]. 

In 2003, interest in VBS was renewed with Medtronic Sofamor Danek’s (Memphis, TN, USA) design of nitinol staples that were shown to be effective at stabilizing curve progression in adolescent idiopathic scoliosis [79]. These staples have straight prongs when inserted but the prongs then clamp into a “C” shape at body temperature, resulting in an improved and more secure fixation [80]. A significant benefit of VBS is that it theoretically preserves spine growth and loss of motion. Indeed, in a porcine model, Bylski-Austrow et al. demonstrated that VBS effectively decreased vertebral body growth plate and disc heights, particularly the hypertrophic zone height and cell width, supporting the concept that compression of the growth plate was the mechanism of growth inhibition [81]. Results equivalent to bracing have been reported when VBS is used to treat small curves in adolescent idiopathic scoliosis. When comparing vertebral body stapling and bracing in adolescents with Risser scores ≤ 1, Cuddihy et al. found an equivalent reduction of 25–34° in the curve progression of the thoracic curves and of 25–34° in that of the lumbar curves between bracing and VBS [82]. Unfortunately, these results have not translated to larger curves. O’Leary et al. found that in 11 adolescents with severe non-idiopathic curves (average 68°), 73% (*n* = 8) of patients required or were considering a second operation at the 2 month follow-up [83]. Thus, VBS is a poor choice for curves >35° but may be an alternative to bracing for children who cannot tolerate such external immobilization [82].

### 3.7. Vertebral Body Tethering

Utilizing a similar conceptual technique to staples, animal studies suggested that surgical manipulation of the anterior vertebral body can alter vertebral body growth and correct scoliosis, while preserving spinal motion [84,85]. Vertebral body tethering (VBT) is an anterior technique that uses cords and vertebral body screw constructs to correct scoliotic curves via growth modulation while maintaining spinal mobility [86,87,88]. A biomechanical study by Nicolini et al. demonstrated that VBT constructs from T11 to L3 preserved the global spinal range of motion in flexion extension and axial rotation [89]. At a minimum two-year follow-up, studies by Miyanji et al. and Newton et al. reported VBT success rates of 57 and 59%, respectively. Clinical success was defined as maintaining a radiographic major coronal curve angle ≤ 30° at follow-up [90,91]. Conversely, in a comparative study of patients with larger curves treated with either posterior spinal fusion or VBT, Newton et al. found that those treated with VBT had significantly worse residual deformity, with a mean thoracic curve of 33° ± 18° versus 16° ± 6° for the posterior spinal fusion group (*p* < 0.001) [92]. Additionally, overall complications were worse as well—with 52% (12/23) having broken tethers and 39% (9/23) patients requiring an entirely separate revision [92]. Other concerns regarding VBT include specific surgical indications, long-term preservation of spinal mobility, postoperative lung function, and quality-of-life. The debate continues as to whether VBT or a more definitive spinal fusion method in children of similar skeletal maturity is more effective and less morbid.

## 4. Conclusions 

The treatment of scoliosis has long been a focus in orthopedics. Surgical devices and techniques have undergone significant evolution over time. In children specifically, implant choices to manage scoliosis are numerous. For children with significant growth remaining, growth-modulated constructs, such as growing rods, vertical expandable prosthetic titanium ribs, and vertebral body tethering can be used to dynamically correct the deformity as the patient matures. For children without significant growth, static fixation methods are abound. Both static and dynamic fixation options often share implant types and techniques, along with devices that fix to the pelvis. Understanding the fixation options available, along with the risks and benefits of each technique and their developmental roots, is important to optimize the surgical outcomes for the scoliosis patient population. This article serves as an overview of both past and present surgical techniques and their outcomes, with the understanding that, given the available literature, we are unable to provide a direct comparison of these treatments.
